# Discovery and validation of a glioblastoma co-expressed gene module

**DOI:** 10.18632/oncotarget.24228

**Published:** 2018-01-13

**Authors:** Leland J. Dunwoodie, William L. Poehlman, Stephen P. Ficklin, Frank Alexander Feltus

**Affiliations:** ^1^ Department of Genetics and Biochemistry, Clemson University, Clemson, SC 29634, USA; ^2^ Department of Horticulture, Washington State University, Pullman, WA 99164, USA

**Keywords:** glioblastoma, systems biology, gene co-expression networks, complement system, cancer

## Abstract

Tumors exhibit complex patterns of aberrant gene expression. Using a knowledge-independent, noise-reducing gene co-expression network construction software called KINC, we created multiple RNAseq-based gene co-expression networks relevant to brain and glioblastoma biology. In this report, we describe the discovery and validation of a glioblastoma-specific gene module that contains 22 co-expressed genes. The genes are upregulated in glioblastoma relative to normal brain and lower grade glioma samples; they are also hypo-methylated in glioblastoma relative to lower grade glioma tumors. Among the proneural, neural, mesenchymal, and classical glioblastoma subtypes, these genes are most-highly expressed in the mesenchymal subtype. Furthermore, high expression of these genes is associated with decreased survival across each glioblastoma subtype. These genes are of interest to glioblastoma biology and our gene interaction discovery and validation workflow can be used to discover and validate co-expressed gene modules derived from any co-expression network.

## INTRODUCTION

Glioblastoma (GBM) tumors, with an adult median survival time of 14.6 months after radiation and temozolomide therapy [1], are known for their heterogeneity, vascularization, and lethality. Even after resection, remaining tumor cells multiply and invade the surrounding parenchyma. Interestingly, primary GBM has few known risk factors [2] -- GBM affects patients across age, cultural, and socioeconomic boundaries. The discovery of relevant biomarker combinations driving GBM tumorgenicity would have therapeutic implications.

There are known monogenic GBM biomarkers that include mutations in the IDH1 [3] and PDGFRα [4] loci. However, any given biomarker does not provide a complete picture of the GBM microenvironment. GBM tumors, as with other tumors, diseases, and complex traits, are controlled by a variety of genetic and epigenetic factors [5]. Thus, a systems approach is needed to fully understand the biology underlying the GBM phenotype. Fortunately, modern measurement technologies such as next-generation sequencing [6] now provide researchers a broad genomics perspective that is revealing new insights to human disease. These technologies, coupled with genomics and epigenomics databases such as The Cancer Genome Atlas (TCGA) [7], used in this investigation, encourage new discoveries.

To identify complex gene expression relationships in multiple human tumors, we have built RNAseq-based gene expression matrices (GEMs) from publicly available RNAseq datasets. One GEM contains gene expression profiles for GBM, lower grade glioma (LGG), bladder urothelial carcinoma (BLCA), thyroid carcinoma (THCA), and ovarian serous cystadenocarcinoma (OV) from TCGA [7] and is fully described in another publication [8]. Another GEM built for this study contains different RNAseq expression profiles for GBM [9], normal brain [10], and Parkinson’s brain [11] obtained from the NCBI SRA archive [12]. Both GEMs were individually preprocessed and transformed into a gene co-expression network (GCN) using Knowledge-Independent Network Construction (KINC) software [8]. Complex gene interactions present in the input samples are mineable from these GCNs, and these gene interactions patterns can be compared between GCNs.

The KINC software package ([8]; open source code available at http://www.github.com/SystemsGenetics/KINC) is unique because it deconvolutes mixed-condition expression patterns (e.g. mixed tumor expression profiles in the GEM), allowing significant co-expression relationships to be annotated with sample labels (e.g. GBM vs. non-GBM). There is no need to separate gene expression profiles prior to analysis. Thus, without providing KINC *a priori* knowledge, tumor- or non-tumor-specific gene expression relationships can be identified and analyzed for biological meaning.

In this report, we apply a generalizable gene interaction discovery and validation workflow, outlined in Supplementary Figure 1, that allows for the detection of condition-specific gene sets in one GCN that can be validated for specificity and reproducibility in alternate GCNs. We focused this approach on GBM-specific modules to investigate GBM tumor biology. Herein, we describe the discovery and characteristics of a GBM-specific gene module present in two GCNs, and explore the expression patterns and epigenetic state of these genes across the spectrum of GBM subtypes using multiple *in silico* approaches.

## RESULTS AND DISCUSSION

### GCN construction

**Figure 1:**
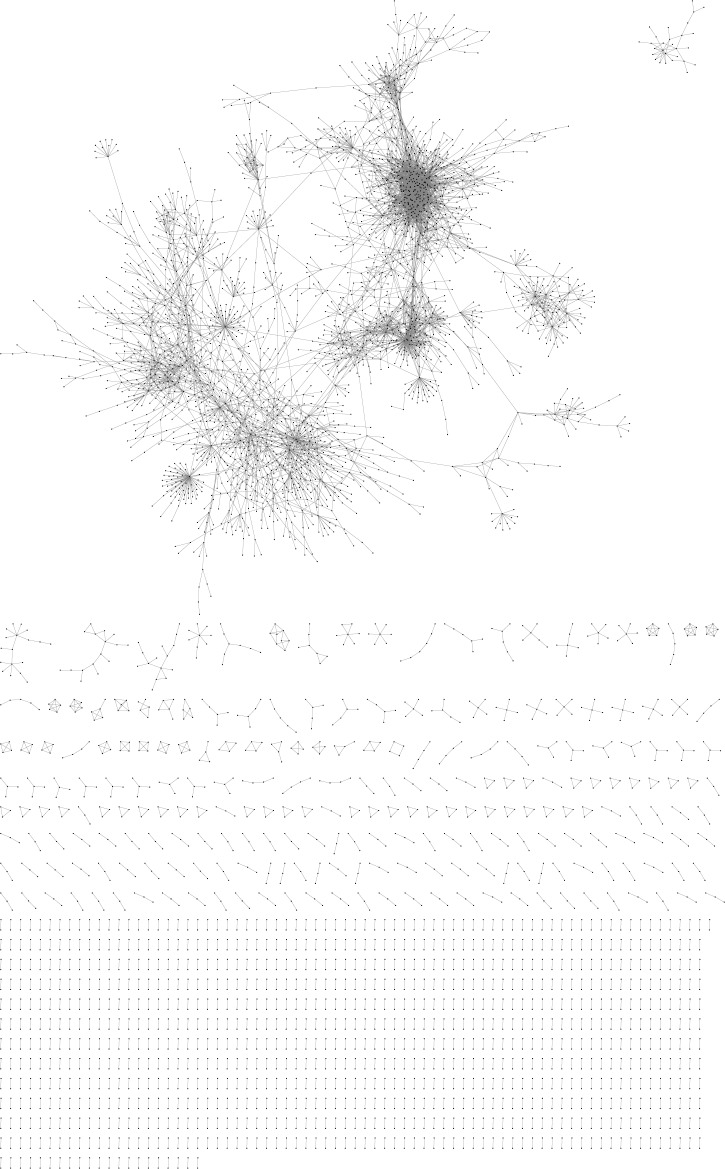
Visualization of the brain network Points represent transcripts (nodes) and lines represent significant expression correlations (edges) between nodes.

KINC identified significant co-expression relationships among 2016 datasets in the TCGA Network and among 204 datasets in the Brain Network. The TCGA network, obtained from our previous study in Ficklin *et al.* [8], included GBM and LGG datasets along with BLCA, OV, and THCA datasets. The Brain Network included GBM and normal brain datasets along with datasets from Brodmann’s Area 9 of Parkinson’s patients [11]. The TCGA Network (Supplementary Table 1) is described in [8] and the Brain Network (Supplementary Table 2) was visualized with Cytoscape [41] and shown in Figure 1. 356 LCMs were detected in the TCGA Network ([8]; Supplementary Table 3) and 456 LCMs were found in the Brain Network (Supplementary Table 4). Many of these modules were condition-specific; for example, 68 of the Brain Network’s 456 modules were enriched for GBM (*p <* 0.001).

### Gene module discovery

Genes in the Brain Network were compared with those in the TCGA Network to investigate commonality in GBM co-expression. 477 unique genes -- 6.6% of the unique genes in the TCGA Network and 8.9% of the unique genes in the Brain Network-were found in both networks. In addition, each network was parsed into LCMs. The TCGA Network had 356 modules total and the Brain Network had 456 modules total. Considering only the genes in these modules, 74 unique genes mapped between networks. The low amount of overlap may be a function of (A) sparse gene overlap between GCNs; and (B) differential Type I and Type II error in each GCN partially due to alternate expression measurement techniques; the TCGA Network used a GEM made with TCGA’s RNAseqV2 workflow [15] and 73599 knownGene 5 UCSC transcript IDs, while the Brain Network used a GEM made with Hisat [18], Cufflinks [21], and 209086 Ensembl hg38 transcript IDs. Nonetheless, of these 74 overlapping genes, 22 (Table 1) were seen in Modules M0257 (in the Brain Network) and M0214 (in the TCGA Network). While the fate of other common interactions is unclear, these 22 intersecting GBM interactions emerged in both GCNs.

**Table 1:** The 22 genes overlapping between TCGA M0214 and brain M0257

Gene Symbol	Gene Name	hg38 Ensembl ID	kg5 UCSC ID	Entrez ID	Chromosome	Transcription Start	Transcription Stop	Gene Friends
LAPTM5	lysosomal protein transmembrane 5	ENST00000294507	uc002iop.1	7805	1	30732469	30757820	19
C1QA	complement C1q A chain	ENST00000374642	uc001bfy.2	712	1	22636506	22639608	10
FCER1G	Fc fragment of IgE receptor Ig	ENST00000367992	uc001bga.3	2207	1	161215279	161220699	22
C1QC	complement C1q C chain	ENST00000374639	uc001qtv.1	714	1	22643633	22648110	11
CD86	CD86 molecule	ENST00000330540	uc002jkv.2	942	3	122055374	122121136	22
HAVCR2 (TIM-3) [23]	hepatitis A virus cellular receptor 2	ENST00000307851	uc003eet.2	84868	5	157085832	157109714	21
LY86	lymphocyte antigen 86	ENST00000379953	uc001fyz.1	9450	6	6588108	6654983	16
TREM2	triggering receptor expressed on myeloid cells 2	ENST00000373113	uc001nym.2	54209	6	41158507	41163176	9
FERMT3	fermitin family member 3	ENST00000345728	uc001xvv.2	83706	11	64206734	64223886	19
SPI1	Spi-1 proto-oncogene	ENST00000378538	uc003lwk.1	6688	11	47354860	47378576	19
C3AR1	complement C3a receptor 1	ENST00000307637	uc002zgf.3	719	12	8058302	8066471	21
GPR65	G protein-coupled receptor 65	ENST00000267549	uc001bsc.2	8477	14	88005124	88014811	17
RNASE6	ribonuclease A family member k6	ENST00000304677	uc003mwy.1	10048	14	20781051	20782467	22
ABI3	ABI family member 3	ENST00000225941	uc002mkg.2	51225	17	49210227	49223225	21
CD300A	CD300a molecule	ENST00000360141	uc011aqf.1	11314	17	74466416	74484796	19
TYROBP	TYRO protein tyrosine kinase binding protein	ENST00000262629	uc001vye.3	7305	19	35904410	35908295	22
SIGLEC9	sialic acid binding Ig like lectin 9	ENST00000250360	uc004euu.2	27180	19	51124908	51130310	19
MYO1F	myosin IF	ENST00000613525	uc002pvu.2	4542	19	8520797	8577577	18
ITGB2	integrin subunit beta 2	ENST00000397852	uc001nfb.1	3689	21	44885953	44910826	22
PARVG	parvin gamma	ENST00000356909	uc003opy.2	64098	22	44181400	44206635	19
WAS	Wiskott-Aldrich syndrome	ENST00000376701	uc002ocm.2	12731	X	48683779	48691427	19
SASH3	SAM and SH3 domain containing 3	ENST00000356892	uc004dkm.3	54440	X	129779984	129795201	19

Next, we asked if there was corroborative co-expression evidence for the 22 interacting genes from other sources. Specifically, we searched the 22 matching genes as a group in the GeneFriends gene co-expression database [27]. Table 1 shows the gene set co-expression analysis where co-expression probability is represented by the GeneFriends binomial cumulative distribution function (*p <* 1.00E-7). Because 22 genes were provided to GeneFriends, each gene has a maximum of 22 friends, or co-expressed genes.

Interestingly, TCGA M0214 (*p <* 2.56E-17) and Brain M0257 (*p <* 1.37E-15) were both enriched for GBM but not LGG (*p <* 4.52E-03 in the TCGA Network) or normal brain (*p <* 1.00 in the Brain Network). TCGA M0214 is also enriched for ovarian cancer (OV; *p <* 2.56E-17). Table 2 shows the condition-specific Fisher’s Exact Test enrichment values for TCGA M0214 and Brain M0257. In total, TCGA M0214 includes 54 genes and Brain M0257 includes 63 genes (Supplementary Table 5). The clinical annotation term enrichment results are available for each TCGA module (Supplementary Table 3) and each Brain module (Supplementary Table 4).

**Table 2:** Condition-specific module enrichment

Network	Module	Condition	Enrichment
TCGA	M0214	GBM	2.56E-17
TCGA	M0214	LGG	4.52E-03
TCGA	M0214	OV	1.61E-14
TCGA	M0214	THCA	1.00E+00
TCGA	M0214	BLCA	1.00E+00
Brain	M0257	GBM	1.37E-15
Brain	M0257	Normal Brain	1.00E+00
Brain	M0257	Parkinson	2.05E-01

It was interesting that TCGA M0214 is enriched for both GBM and OV. While OV is enriched for 57 modules and GBM is enriched for 102 modules (Supplementary Table 3), 22 modules are enriched for both GBM and OV. While these cancers are seemingly very different, further investigations might reveal new commonalities between them. Indeed, the literature shows few links between GBM and OV, but OV can metastasize to the brain [42] and bevacizumab, an angiogenesis inhibitor used to treat GBM, has been found to alleviate mesenchymal-like, proliferative OV subtypes [43]. While TCGA M0282, described in Ficklin *et al.* [8], is also enriched for GBM and OV in addition to THCA, no genes are shared between TCGA M0282 and M0257. Indeed, no genes are shared between TCGA M0282 and the Brain Network.

### Internetwork gene module validation shows GBM-specific correlations

**Figure 2:**
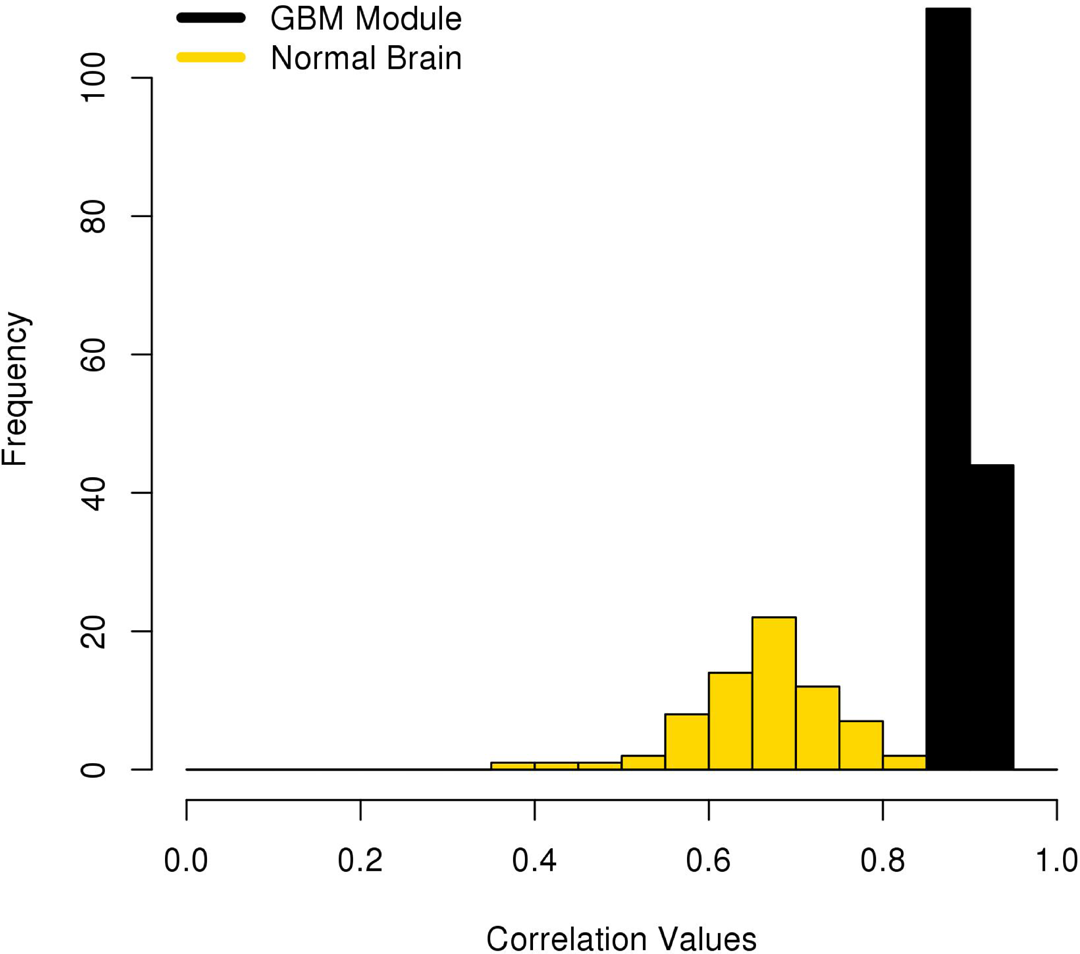
Brain M0257 Correlation values mapped to normal brain datasets The Spearman correlation values of pairwise gene expression are shown for Brain M0257 and matching edges using only normal samples from the Brain GEM.

**Figure 3:**
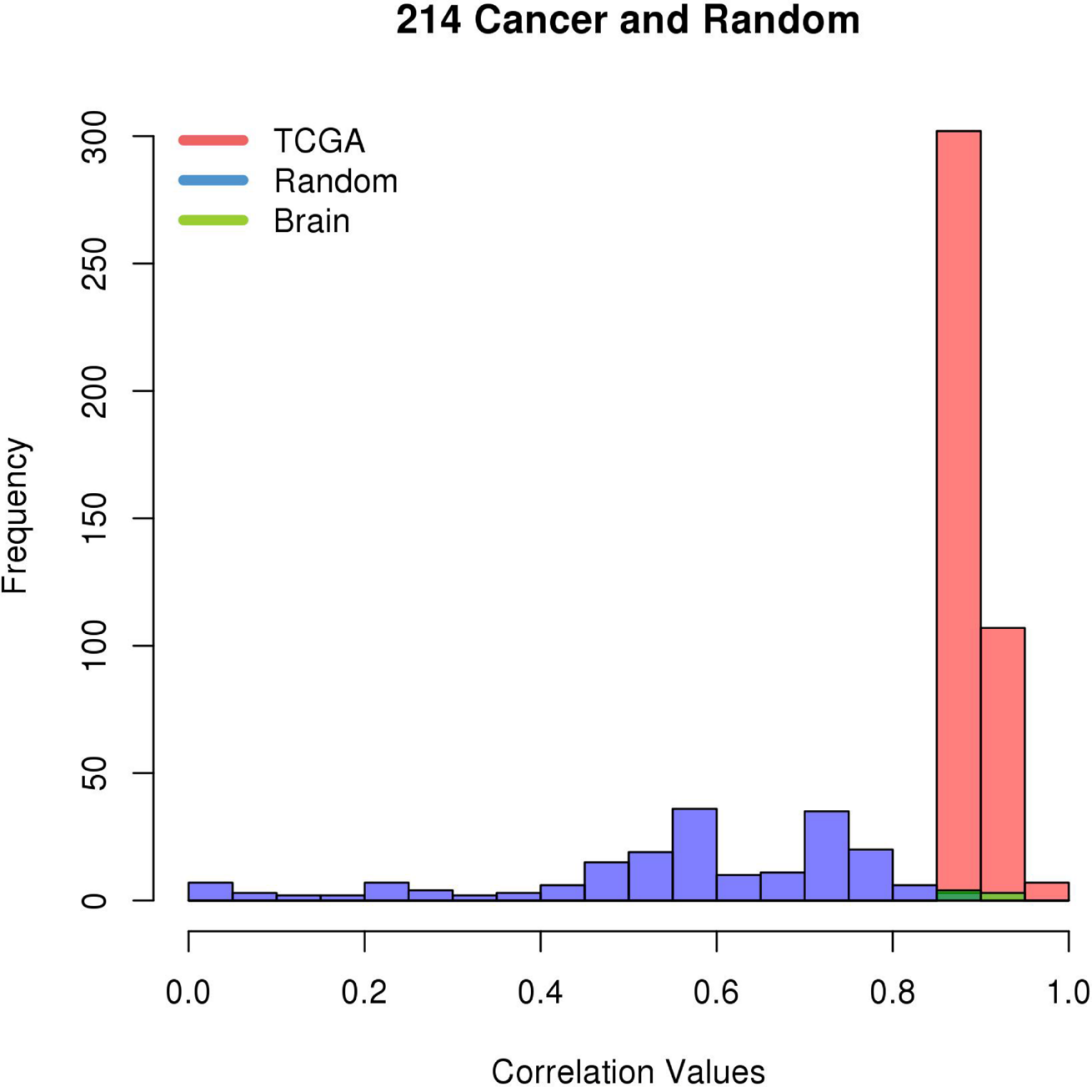
Correlation values from TCGA M0214 mapped to Brain M0257 and Random GEMs The Spearman correlation values of pairwise gene expression are shown for TCGA M0214, Brain M0257, and a miniature GEM created using expression levels from the Random GEM and the genes in TCGA M0214. Only the edges in Brain M0257 that exactly match edges in TCGA M0214 are shown.

The correlations in Brain M0257, which is enriched for GBM, were compared to matching correlations found by KINC using only the normal brain datasets in the Brain GEM. 70 of the 154 edges (45.45%) in Brain M0257 were rediscovered in the normal brain datasets. Of these edges, none had a significant Spearman correlation greater than 0.8801, the Brain Network’s RMT threshold (Figure 2). TCGA M0214 includes 54 unique transcripts and 416 edges correlated above its RMT threshold. 49 of these UCSC kg5 transcripts mapped to the hg38 Ensembl IDs used by the Brain and Random GEMs. As described in the Methods section, the expression values for these 49 transcripts in the Random GEM were processed with KINC [8]. KINC rediscovered 191 (45.91%) of the 416 TCGA edges using expression values from the Random GEM. Nine (4.71%) of the 191 rediscovered edges in the Random GEM had a Spearman correlation greater than 0.7901, the Random Network’s RMT threshold. Seven (4.55%) of the 154 edges in Brain M0257 were also found in TCGA M0214; 100% of these edges have a Spearman correlation greater than the Brain Network’s significance threshold (Figure 3). These data indicate that the pairwise correlation of these genes is GBM-specific.

### Gene expression analysis reveals GBM-specific upregulation

**Figure 4:**
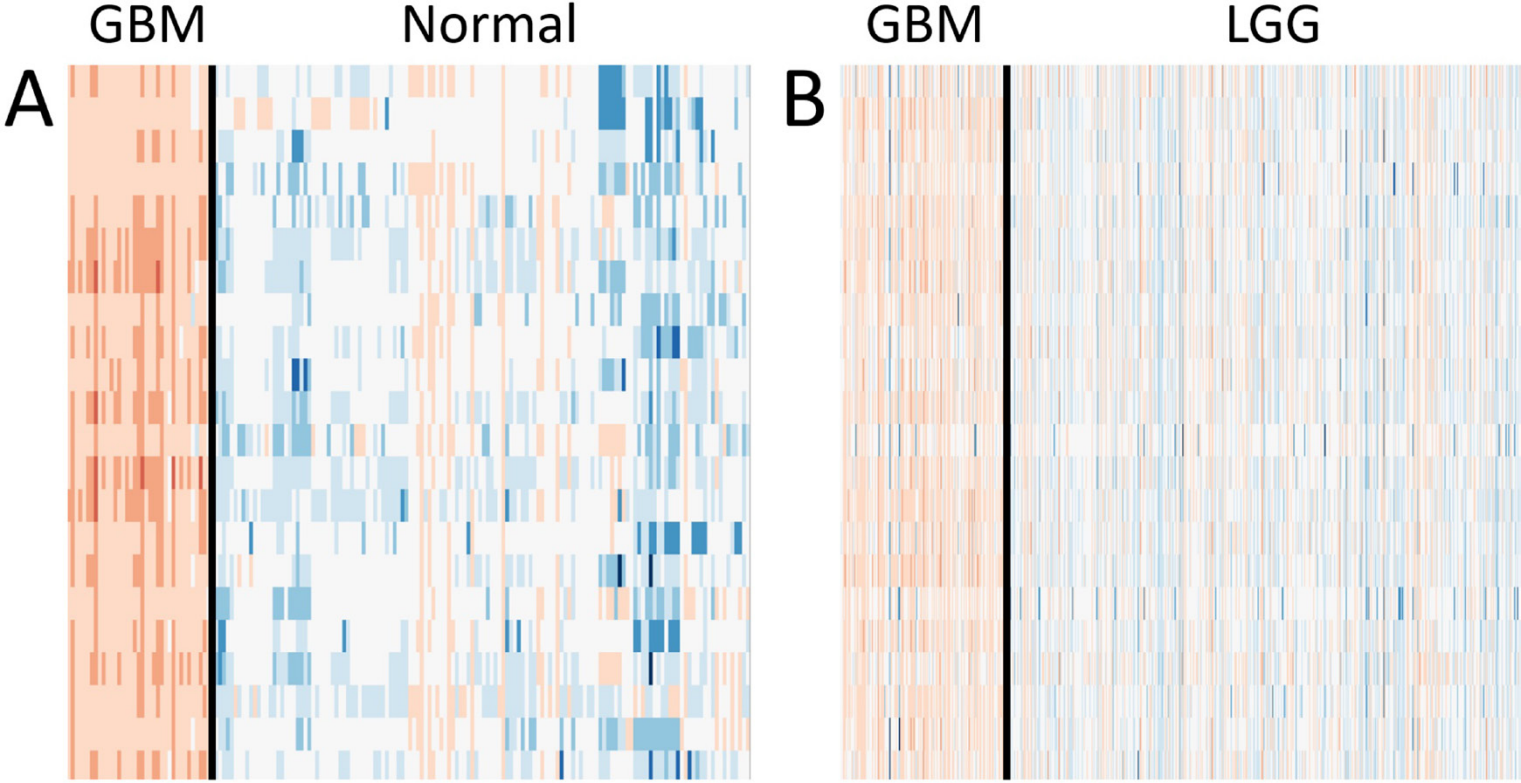
Expression levels of the 22 matching genes **(A**) GBM and normal brain expression levels in the normalized Brain GEM. (**B**) GBM and LGG expression levels in the normalized TCGA GEM. Red indicates expression above the mean, blue indicates expression below the mean, and white indicates expression near the mean.

**Figure 5:**
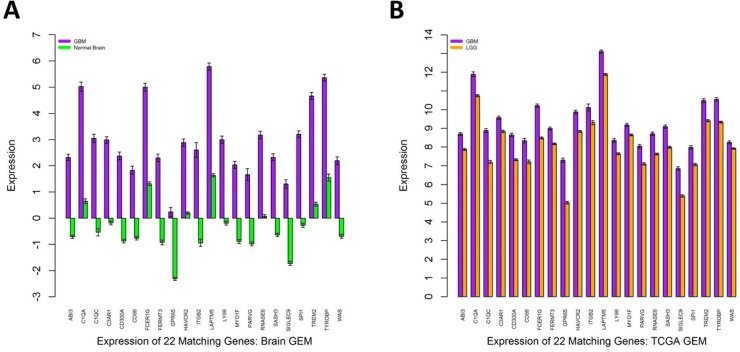
Expression levels of the 22 matching glioblastoma genes **(A**) The expression of the 22 matching genes in the Brain GEM after dividing the normalized expression values by either the GBM or normal brain median expression value. Vertical axis units represent normalized FPKM values. (**B**) The expression of the 22 matching genes in the TCGA GEM. No further normalization was performed. Vertical axis units represent normalized RPKM values. Error bars represent SEM.

We investigated the gene expression levels of the 22 genes seen in TCGA M0214 and Brain M0257 in different conditions. Heatmaps and bar graphs were constructed to visualize the expression levels of the 22 matching genes between TCGA M0214 and Brain M0257 (Figures 4A–4B and 5A–5B). All 22 genes showed significantly upregulated expression (Student’s *T* Test; *p <* 0.001) in GBM relative to LGG (in the TCGA Network) and relative to normal brain (in the Brain Network). Significance test results are available for the expression of the 22 shared genes in the TCGA GEM (Supplementary Table 6) and in the Brain GEM (Supplementary Table 7).

### Putative transcriptional regulators of the GBM modules

Several gbmSygnal biclusters [37] involving genes from TCGA M0214 or Brain M0257 (Supplementary Table 8) implied regulation by the transcription factor ELF1. In addition, by loading the 22 matching genes into the Broad Institute Gene Set Enrichment Analysis [34], we found that ELF1 was the second-most enriched transcription factor for this dataset (*p <* 9.91 × 10^–8^) (Supplementary Table 9). Indeed, 5 of the 22 matching genes have putative ELF1 sites. Moreover, one ELF1 transcript, ENST00000239882, was upregulated in the Brain GEM GBM data relative to normal brain data (*p <* 3.79 × 10^–27^) (Supplementary Table 10). Furthermore, this transcript’s kg5 UCSC counterpart, uc001uxs.2, was also upregulated in the TCGA GEM GBM data relative to LGG data (*p <* 1.94 × 10^–4^) (Supplementary Table 10).

In addition, one of the 22 matching genes, SPI1, is a transcription factor that shares the ETS transcription factor family with ELF1. The 22 matching genes were also provided to the transcription factor function in the GeneFriends database [27] (Supplementary Table 11). Of 1538 possible transcription factors, SPI1 was the 5th-most enriched for the gene set (*p <* 1.01 × 10^–23^) and 19 of the 22 genes were co-expressed with SPI1. In addition, the 22 matching genes were provided to the RegNetwork database [35] (Supplementary Table 12). SPI1 was the only one of the 22 matching genes to be considered a regulator by RegNetwork. Eight of the 21 other genes, three with high confidence, were considered regulated by SPI1. Indeed, of the 2221 genes potentially regulated by SPI1, three of the 22 genes ranked very highly-ITGB2 was ranked 3rd, WAS 5th, and TYROBP 27th.

### Internetwork modular methylation analysis shows GBM-specific hypo-methylation

**Figure 6:**
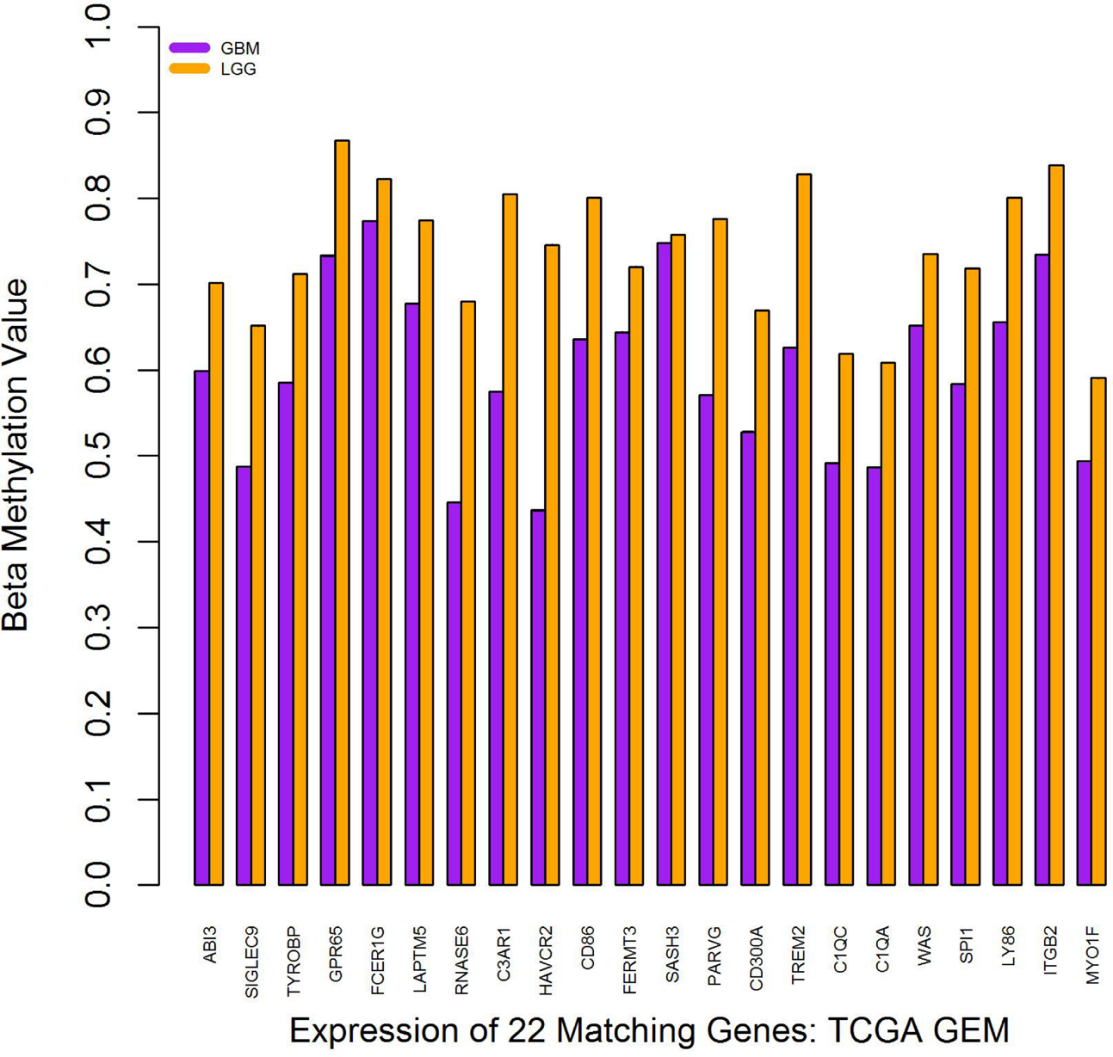
Beta methylation values for the 22 matching glioblastoma genes Error bars show SEM. Note that, because there are several methylation readings for each dataset and hundreds of datasets per condition, no error bars are visible because the SEM is appreciably zero.

The beta methylation values for each of the 22 matching genes were evaluated using data from TCGA. A Student’s *T*-Test was used to compare the beta methylation values for LGG with those in GBM (*p <* 6.84 × 10^–6^). As shown in Figure 6, on average, each of the 22 genes was hypo-methylated in GBM relative to LGG. Of interest, while TCGA M0214 is enriched for GBM and OV, the methylation patterns differ between GBM and OV. While these 22 genes are hypo-methylated in GBM versus LGG datasets, several of these 22 genes are hyper-methylated in OV versus LGG datasets (data not shown), suggesting an alternate regulatory mechanism in OV.

### GBM subtype analysis supports the mesenchymal phenotype

**Figure 7:**
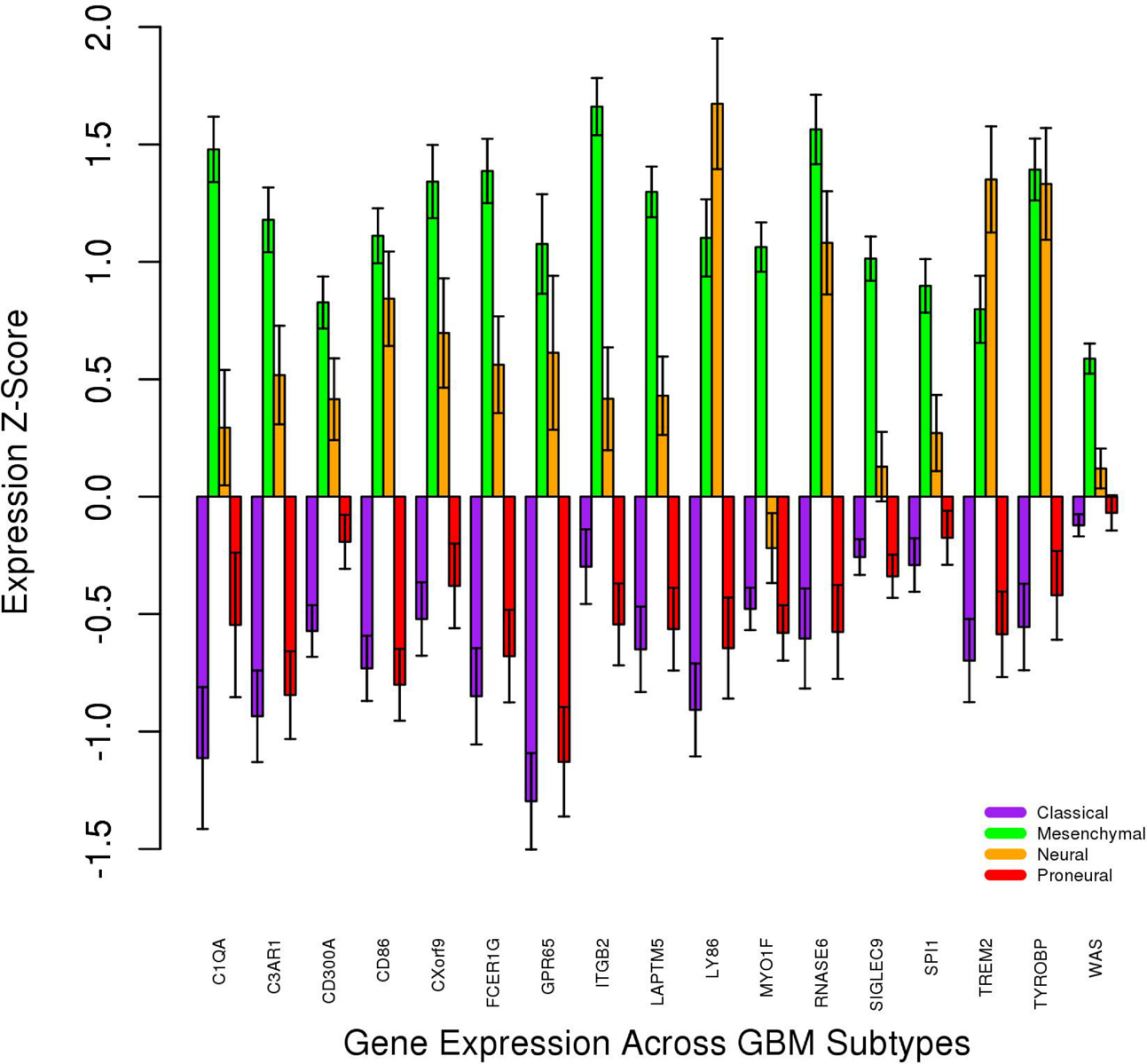
Gene expression levels across GBM subtypes for 17 of the 22 matching genes Error bars represent SEM.

The gbmSygnal Network [37] uses bicluster technology to group genes based on ChIPseq signals and gene co-expression. Each of the 22 matching genes was searched in the gbmSygnal database. 16 biclusters enriched for a cancer hallmark were found with three or more of the 22 matching genes. These 16 biclusters were all enriched for “tumor-promoting inflammation” and “evading immune detection” (Supplementary Table 8). In addition, the gbmSygnal Network organizes expression data for each bicluster into quintiles and enriches each quintile for GBM subtype. Mesenchymal GBM was predominant in the highest expression quintile relative to the lowest expression quintile in each of the 16 biclusters. Furthermore, Verhaak *et al.* [44] described four subtypes of GBM [44] and genes upregulated in each of the four GBM subtypes. Four genes (SIGLEC9, MYO1F, LAPTM5, ITGB2) from the list of 22 matching genes were upregulated in mesenchymal GBM; none of the 22 genes were upregulated in any other subtype. Furthermore, we investigated the expression levels of nine NF1 transcripts in the Brain GEM. High NF1 expression is characteristic of mesenchymal GBM [44]. One of these transcripts, ENST00000358273, was upregulated in GBM relative to normal brain (Student’s *T*-Test *p*-value = 6.56 × 10^–15^; Supplementary Table 13). Finally, 17 of the 22 shared genes were found in the Glioblastoma Bio Discovery Portal [38] based on results from Verhaak *et al.* [44]. The average mRNA expression z-score was found across the proneural (*n =* 56 tumors), neural (*n =* 31 tumors), mesenchymal (*n =* 57 tumors), and classical (*n =* 53 tumors) GBM subtypes (Figure 7). The mesenchymal subtype showed the highest expression for 15 of 17 genes. Furthermore, using the Glioblastoma Bio Discovery Portal, it was found that above-median expression levels for these 17 genes led to decreased survival in every GBM subtype and the full cohort (Table 3).

**Table 3:** Glioblastoma bio discovery portal survival analysis

Subtype	Prognostic index hazard ratio	LogRank *P*-Value
Classical	3.57	0
Neural	15.63	0
Proneural	4.66	0
Mesenchymal	3.02	0
Full Cohort	1.79	0

Prognostic index hazard ratio and logrank *p*-value are given for expression levels above the median within each subtype.

### cBioPortal analysis provides evidence for a GBM-specific module

If this 22-gene network is GBM-specific, as shown in Figures 4B and 5B, one would expect different co-occurrence and mutual exclusivity results for LGG and GBM data (Table 4). Indeed, this 22-gene network appears highly dependent on ATRX and p53 in LGG but not in GBM. The mutual exclusivity of PIK3R1 mutations in GBM is of particular interest; PIK3R1 knockdown decreases invasion, proliferation, and migration in GBM [45]. IDH1 mutations were neither mutually exclusive nor co-occurring with alterations in the 22 shared genes. This is consistent with Figure 7; the proneural GBM subtype, which exhibits low expression for these 22 genes, is often defined by IDH1 or PDGFRA mutation.

**Table 4:** Mutation enrichment in shared genes

Condition	Gene	Direction	Enrichment
LGG	ATRX	Co-occurrence	2.56E-06
LGG	TP53	Co-occurrence	5.04E-06
LGG	CIC	Mutual exclusivity	1.29E-03
LGG	MUC17	Co-occurrence	0.0122
LGG	FUBP1	Mutual exclusivity	0.0143
LGG	VPS13B	Co-occurrence	0.019
LGG	MYH8	Co-occurrence	0.029
GBM	KCNQ5	Co-occurrence	3.84E-03
GBM	MUC17	Co-occurrence	0.0126
GBM	LILRB2	Co-occurrence	0.016
GBM	PTPRM	Co-occurrence	0.016
GBM	PIK3R1	Mutual exclusivity	0.0326

### Internetwork modular functional annotation analysis

We next sought to understand the function of TCGA M0214 and Brain M0257 in GBM. Comparing the functional annotations enriched (*p <* 0.001) in TCGA M0214 and Brain M0257 showed that 23 annotations were shared between modules (Table 5). C1QA and C1QC, components of complement protein C1 [46], and C3AR1, a receptor for the complement protein C3a [47], are among the 22 shared genes between TCGA M0214 and Brain M0257. C1Q has been shown [48] to promote GBM invasiveness and proliferation independent of complement system activation. Carro *et al.* also lists C1Q as a member of the transcriptional network which drives mesenchymal phenotypes in brain tumors [49]. Several complement system-related functional annotations (Table 5) are also shared between TCGA M0214 and Brain M0257. In addition, RNase6, one of the 22 matching genes, was searched with ImmuNet, a regulatory network database for immune system-related genes [36]. At a confidence level > 99%, eight of the other 21 genes shared a function with RNase6 in the complement system (Supplementary Table 14).

**Table 5:** Shared functional annotations in between modules

Term	Term ID	Function
GO	GO:0045087	Innate immune responses are defense responses mediated by germline encoded components that directly recognize components of potential pathogens.
GO	GO:0050776	Any process that modulates the frequency, rate or extent of the immune response, the immunological reaction of an organism to an immunogenic stimulus.
GO	GO:0045650	Any process that stops, prevents, or reduces the frequency, rate or extent of macrophage differentiation.
GO	GO:0005581	A protein complex consisting of three collagen chains assembled into a left-handed triple helix.
GO	GO:0030853	Any process that stops, prevents, or reduces the frequency, rate or extent of granulocyte differentiation.
GO	GO:0006955	Any immune system process that functions in the calibrated response of an organism to a potential internal or invasive threat.
GO	GO:0034138	Any series of molecular signals generated as a consequence of binding to toll-like receptor 3.
GO	GO:0002283	The change in morphology and behavior of a neutrophil resulting from exposure to a cytokine, chemokine, cellular ligand, or soluble factor, leading to the initiation or perpetuation of an immune response.
GO	GO:0002281	A change in morphology and behavior of a macrophage resulting from exposure to a cytokine, chemokine, cellular ligand, or soluble factor, leading to the initiation or perpetuation of an immune response.
GO	GO:0019864	Interacting selectively and non-covalently with an immunoglobulin of an IgG isotype.
GO	GO:0071404	Any process that results in a change in state or activity of a cell (in terms of movement, secretion, enzyme production, gene expression, etc.) as a result of a low-density lipoprotein particle stimulus.
INTERPRO	IPR001073	C1q domain
INTERPRO	IPR008983	Tumour necrosis factor-like domain
INTERPRO	IPR008160	Collagen triple helix repeat
INTERPRO	IPR013106	Immunoglobulin V-set domain
KEGG	hsa05322	Systemic lupus erythematosus
MIM	120575	COMPLEMENT COMPONENT 1, q SUBCOMPONENT, C CHAIN
PFAM	PF00386	C1q is a subunit of the C1 enzyme complex that activates the serum complement system.
PFAM	PF01391	Members of this family belong to the collagen superfamily.
PFAM	PF07686	This domain is found in antibodies as well as neural protein P0 and CTL4 amongst others.
REACTOME	R-HSA-173623	Classical antibody-mediated complement activation
REACTOME	R-HSA-198933	Immunoregulatory interactions between a Lymphoid and a non-Lymphoid cell
REACTOME	R-HSA-166663	Initial triggering of complement

In conclusion, we used a GMM-based gene co-expression analysis to identify GBM-specific gene co-expression clusters embedded within and parsed from LGG and normal brain datasets. A 22-gene module was separately identified in two gene expression sources; this module has increased RNA expression and decreased DNA methylation in GBM. Furthermore, high expression of these genes is associated with decreased survival and with the mesenchymal GBM subtype. Future work involving these genes may help assess their roles in the complex GBM phenotype. We present this GBM-specific gene module and note that the cross-validating co-expression workflow used here is widely applicable.

## MATERIALS AND METHODS

### TCGA GEM construction

As described in Ficklin *et al.* [8], the TCGA GEM was constructed using 2016 RNAseq tumor samples [7]. All available normalized isoform datasets, produced by TCGA’s RNASeqV2 workflow [14, 15], were downloaded for five cancers on April 1, 2016. The datasets include 173 GBM samples, 534 lower grade glioma (LGG) samples, 427 bladder cancer (BLCA) samples, 309 ovarian cancer (OV) samples, and 572 thyroid cancer (THCA) samples. These datasets were compiled into a single GEM, which is an *n x m* matrix where *n* is the number of datasets and *m* is the number of RNA transcript IDs; each value represents a gene’s expression level as quantified by RSEM through the RNASeqV2 workflow [14, 15]. The TCGA data utilized 73599 knownGene version 5 (kg5) UCSC transcript IDs. As such, the raw GEM was a 73599 × 2016 matrix. Outlier expression profiles were detected using a Kolmogorov-Smirnov (KS) test (D_N_ > 0.15) implemented in the *preprocessCore* [16] library. No outliers were detected. All non-zero expression values were log2 transformed and the matrix was quantile normalized.

### Brain and random GEM construction

A brain-specific GEM was constructed using 220 RNAseq datasets from NCBI’s SRA database [12]. These 220 samples were the only publicly-available samples annotated as brain-specific in the SRA database upon their download on September 16, 2016. These datasets were processed into a GEM using the SRA toolkit v2.5.2 [12], Trimmomatic v0.33 [17], Hisat2-2.0.1-beta [18], Samtools v0.1.19 [19, 20], and Cufflinks v2.2.1 [21]. The Gencode v24 GFF3 file, complete with scaffolds, assembly patches, and alternate loci guided transcript quantification (http://www.gencodegenes.org/releases/24.html). The raw GEM was preprocessed through the methods described above. 16 datasets were removed by the KS test (D_N_ > 0.15), leaving 204 samples in the log2 normalized GEM. Transcript counts were indexed as 209086 Ensembl hg38 transcript IDs resulted in a 209086 × 204 GEM. Of these samples, 38 were GBM tumor samples [9], 138 were normal brain samples [10], and 28 were from Brodmann’s Area 9 of Parkinson’s Disease patients [11]. A random human GEM was also constructed using 2004 human RNAseq datasets from the NCBI SRA database. These samples were randomly selected from all available paired-end human RNAseq datasets that were produced by an Illumina HiSeq sequencer. This GEM was constructed and preprocessed as described above. The KS test (D_N_ > 0.15) removed 211 datasets, resulting in a 209086 × 1793 GEM. From this 209086 × 1793 preprocessed GEM, 49 transcripts mapped to the genes present in Module 0214 identified in the TCGA Network.

### GCN construction and thresholding

Each normalized GEM was processed with KINC v1.0 [8], a software package that uses Gaussian mixture models (GMMs) before applying pairwise correlation analyses. For each GEM, the OSG-KINC (https://github.com/feltus/OSG-KINC) workflow was utilized to build a similarity matrix using the KINC software. This workflow utilizes the Pegasus Workflow Management System [22] to execute GMM clustering and pairwise spearman correlation on the Open Science Grid (https://www.opensciencegrid.org). By using GMMs prior to each pairwise comparison, KINC [8] samples clusters that result in co-expressed genes. Only clusters spanning 30 or more samples were further processed with Spearman correlations because a Pearson’s power analysis found that 30 samples resulted in a false positive rate at α = 0.05, a false negative at β = 0.2, and an effect size of 0.5. The Brain and TCGA Networks each took about one month to construct with KINC. Globus [24] was used to transfer KINC output files to the Palmetto Cluster at Clemson University (https://www.palmetto.clemson.edu/palmetto/). Random Matrix Thresholding (RMT) [25] was used to find a correlation significance threshold for each similarity matrix produced by KINC. The thresholding process ignored clusters with low expression levels (< 0.1 FPKM) and/or less than 30 datasets. Correlations above this experimentally determined significance threshold were extracted for each GCN. The TCGA Network, as described in Ficklin *et al.* [8], had a correlation threshold of 0.8601 and the Brain Network had a correlation threshold of 0.8801. Each edge in a GCN represents a relationship between two genes with a correlation value greater than the RMT-defined significance threshold. Link Community Modules (LCM), or groups of co-expressed genes, were identified using the *linkcomm* R package [13, 26].

### TCGA transcript ID mapping

Biomart (http://useast.ensembl.org/biomart) was used to map each hg38 Ensembl transcript ID in the Brain Network to its corresponding hg38 Associated Gene Name. Using the UCSC hg19 database, the kg5 UCSC transcript IDs in the TCGA Network were mapped to kg6 and kg7 UCSC IDs. Using the UCSC hg38 database, these kg7 UCSC IDs were mapped to kg8 UCSC IDs, kg9 UCSC IDs, and finally to hg38 Ensembl IDs. Biomart was then used to map each hg38 Ensembl ID to its corresponding Associate Gene Name. 90% of the original kg5 UCSC IDs mapped to hg38 Ensembl IDs.

### GeneFriends co-expression validation

The 22 matching genes between TCGA M0214 and Brain M0257 were searched as a group in the GeneFriends database [27]. The transcription factor data and the internal co-expression data for the matching genes were downloaded.

### Module enrichment analysis

Each module was tested for sample label enrichment (Fisher’s Exact Test *P* < 0.001; e.g. LGG, GBM, OV, THCA, BLCA, Normal Brain, and Parkinson’s Brain. Only samples present in > 95% of the edges in a module were considered. Functional term enrichment was performed to associate each module with these functional annotations: Kyoto Encyclopedia of Genes and Genomes (KEGG) [28], Gene Ontology (GO) [29], Reactome [30], InterPro [31], Pfam [32], and Mendelian Inheritance in Man (MIM) [33]. A Fisher’s Exact Test *p*-value < 0.001 was considered significant.

### Internetwork comparisons

For GBM comparisons with the Brain GEM, 63 transcripts in Brain M0257 were used to extract a 63 × 138 GEM with only normal brain datasets-no GBM datasets. This mini-GEM was processed with KINC [8] and edges matching those in Brain M0257 were identified. For cancer-specific comparisons with the random GEM, 54 transcripts in TCGA M0214 were identified in the Brain GEM and the Random GEM. 49 of the 54 kg5 UCSC transcripts in the TCGA GEM mapped to hg38 Ensembl transcripts in the Brain and Random GEMs. These 49 transcripts and their expression values were extracted into a 49 × 1793 mini-GEM that was converted into a GCN with KINC. Edges matching those in TCGA M0214 were identified. For the 22 matching genes between TCGA M0214 and Brain M0257, the normalized GBM and LGG expression values in the TCGA GEM were compared with a Student’s *T*-Test. The GBM and normal brain expression values in the Brain GEM were also compared with a Student’s *T*-Test. The Matlab *clustergram* function was used to generate heatmaps that represent gene expression intensities. We recognized that the Brain GEM, created from various publicly available RNAseq datasets, required further normalization within conditions; unlike the TCGA datasets, the Brain datasets were not all derived by the same research network using the same RNASeqV2 workflow. Thus, GBM expression values of the 22 matching genes were divided by the GBM global median expression value in the Brain GEM (1.1398). Similarly, the normal brain expression values of the 22 matching genes were divided by the normal brain global median expression value in the Brain GEM (1.7215). A Student’s *T*-Test was also used to compare ELF1 and NF1 expression levels across different conditions in the TCGA and Brain GEMs.

### Module DNA methylation analysis

All publicly available beta methylation datasets for GBM and LGG were downloaded from the Genomic Data Commons’ TCGA Data Portal (https://gdc-portal.nci.nih.gov/) on January 27, 2017. 1607 datasets, including 534 LGG and 450 GBM datasets, were downloaded. The beta methylation values for the 22 matching genes across each cancer were compared with a Student’s *T*-Test.

### External evidence for internetwork module relationships

The 22 genes matching between TCGA M0214 and Brain M0257 were queried in the Gene Set Enrichment Analysis database [34] and the resulting transcription factor data was downloaded. These 22 genes were also provided to the RegNetwork database [35] to find possible regulatory mechanisms. In addition, RNase6, one of the 22 matching genes, was searched in the ImmuNet database [36] to find functional similarities to genes in the complement cascade. The genes with ImmuNet confidence levels > 0.99 were downloaded. The gbmSygnal Network [37] was also queried and 16 biclusters with three or more of the 22 matching genes were discovered. The 22 matching genes were also provided to the Glioblastoma Bio Discovery Portal [38] using the “Verhaak Core” participants option and the “3-Platform Aggregates” experiment option. Finally, cBioPortal [39, 40] was used to analyze the 22 matching genes in patient samples; the 22 matching genes were queried twice; first, as a gene set using LGG (*n =* 283 tumors) data, and second, using GBM (*n =* 136 tumors) data from the “TCGA, Provisional” dataset. cBioPortal uses a Student’s *T*-Test for its reverse phase protein array comparison and a Fisher Exact Test for mutation enrichment.

## SUPPLEMENTARY MATERIALS FIGURE AND TABLES































## Abbreviations

GBM: glioblastoma, also known as glioblastoma multiforme
LGG: lower grade glioma
BLCA: bladder urothelial carcinoma
THCA: thyroid carcinoma
OV: ovarian serous cystadenocarcinoma
GEM: gene expression matrix
GCN: gene co-expression network
TCGA: the Cancer Genome Atlas
KINC: Knowledge-Independent Network Construction
kg: knownGene
OSG: Open Science Grid
GMM: Gaussian mixture model
RMT: Random Matrix Thresholding
LCM: Link Community Module
