# Supplementary material for: Discovery and validation of a glioblastoma co-expressed gene module
Source: Oncotarget. 2018 Jan 13;9(13):10995–1008. doi: 10.18632/oncotarget.24228 (PMC5834250; doi:10.18632/oncotarget.24228)
Supplement: Supplementary file 1 [file oncotarget-09-10995-s001.pdf]

Discovery and validation of a glioblastoma co-expressed gene module

SUPPLEMENTARY MATERIALS

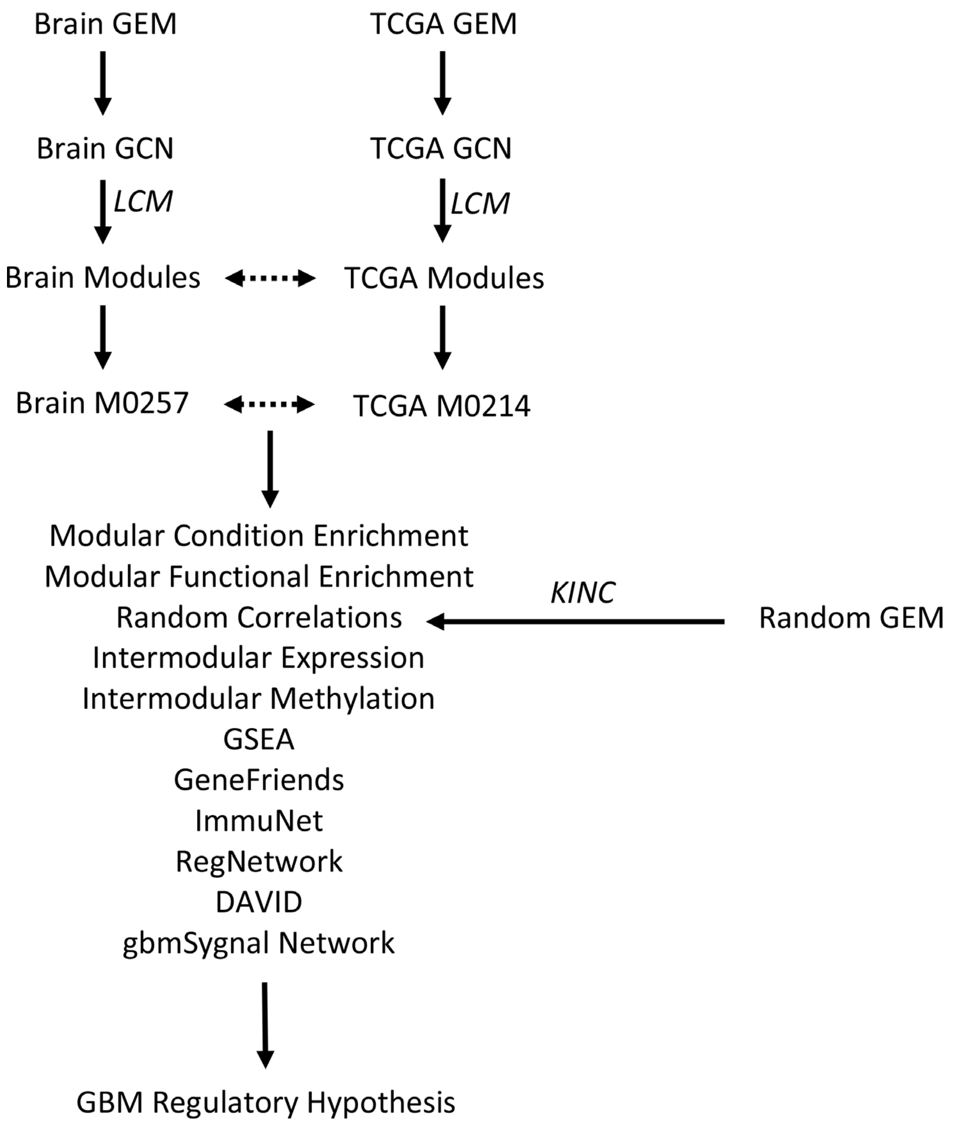

Supplementary Figure 1: Condition-specific gene module discovery and validation workflow. The Brain and TCGA GEMs were each used to create a GCNs and modules with KINC and link communities software, respectively. The genes in these LCMs were compared between GCNs. Two modules, TCGA M0214 and Brain M0257, had 22 overlapping genes. Further analyses compared these modules and their 22 overlapping genes. Dashed double-headed arrows represent these comparisons. Gene expression correlations in random human RNAseq datasets were also compared to these modules. The resulting data was used to hypothesize a common role for TCGA modules M0214 and M0257 in glioblastoma tumors.

Supplementary Table 1: TCGA network edge list. See Supplementary_Table_1

Supplementary Table 2: Brain network edge list. See Supplementary_Table_2

Supplementary Table 3: TCGA network module enrichments. See Supplementary_Table_3

Supplementary Table 4: Brain network modules enrichments. See Supplementary_Table_4

Supplementary Table 5: TCGA M0214 and brain M0257 genes. See Supplementary_Table_5

Supplementary Table 6: Gene expression of the 22 shared genes in the TCGA GEM. See Supplementary_Table_6

Supplementary Table 7: Gene expression of the 22 shared genes in the Brain GEM. See Supplementary_Table_7

Supplementary Table 8: gbmSygnal biclusters involving genes from TCGA M0214 or Brain M0257. See Supplementary_Table_8

Supplementary Table 9: Transcription factor enrichment. See Supplementary_Table_9

Supplementary Table 10: ELF1 transcription factor enrichment. See Supplementary_Table_10

Supplementary Table 11: GeneFriends analysis. See Supplementary_Table_11

Supplementary Table 12: RegNetwork database analysis. See Supplementary_Table_12

Supplementary Table 13: GBM versus normal expression analysis. See Supplementary_Table_13

Supplementary Table 14: ImmuNet database analysis. See Supplementary_Table_14
